# Landraces of snake melon, an ancient Middle Eastern crop, reveal extensive morphological and DNA diversity for potential genetic improvement

**DOI:** 10.1186/s12863-018-0619-6

**Published:** 2018-05-23

**Authors:** Samer Omari, Yuri Kamenir, Jennifer I. C. Benichou, Sarah Pariente, Hanan Sela, Rafael Perl-Treves

**Affiliations:** 1 Mina and Everard Goodman Faculty of Life Sciences, Bar Ilan University, 5290002 Ramat Gan, Israel; 2 Department of Geography and Environment, Bar Ilan University, 5290002 Ramat Gan, Israel; 3 Cereal Crop Improvement Institute, Faculty of Life Sciences, Tel-Aviv University, 6997801 Tel Aviv, Israel

**Keywords:** *Cucumis melo*, Snake melon, Germplasm, Population structure, Genetic diversity, Landraces

## Abstract

**Background:**

Snake melon (*Cucumis melo* var. *flexuosus*, “Faqqous”) is a traditional and ancient vegetable in the Mediterranean area. A collection of landraces from 42 grower fields in Israel and Palestinian territories was grown and characterized in a “Common Garden” rain-fed experiment, at the morphological-horticultural and molecular level using seq-DArT markers.

**Results:**

The different landraces (“populations”) showed extensive variation in morphology and quantitative traits such as yield and femaleness, and clustered into four horticultural varieties. Yield was assessed by five harvests along the season, with middle harvests producing the highest yields. Yield correlated with early vigor, and with femaleness, but not with late vigor. At the molecular level, 2784 SNP were produced and > 90% were mapped to the melon genome. Populations were very polymorphic (46–72% of the markers biallelic in a 4 individuals sample), and observed heterozygosity was higher than the expected, suggesting gene flow among populations and extensive cross pollination among individuals in the field. Genetic distances between landraces were significantly correlated with the geographical distance between collecting sites, and with long term March precipitation average; variation in yield correlated with April temperature maxima.

**Conclusions:**

The extensive variation suggests that selection of local snake melon could result in yield improvement. Correlations between traits and climatic variables could suggest local adaptation of landraces to the diverse environment in which they evolved. This study stresses the importance of preserving this germplasm, and its potential for breeding better snake melons as an heirloom crop in our region.

**Electronic supplementary material:**

The online version of this article (10.1186/s12863-018-0619-6) contains supplementary material, which is available to authorized users.

## Background

Local landraces of crop plants represent an invaluable genetic resource for breeding in a changing environment. Landraces exhibit fine adaptation to the specific environment in which they have evolved under domestication. They often harbor rich genetic diversity, important for traditional agriculture sustainability, that has been eroded in modern cultivars [1, 2]. It is therefore important to generate germplasm collections, describe landraces thoroughly and evaluate their diversity.

Israel is located on the border of three different phyto-geographical zones: the Mediterranean, Saharo-Arabian, and Irano-Turanian zones, respectively [3]. Over very short distances there are notable differences in rainfall, temperature regimes and soil types. In addition, yearly fluctuations in climatic parameters are large, and such instability has been proposed to favor genetic diversity, compared to very stable environments [4, 5]. In addition to natural factors, domestic landraces are also affected by active human agricultural practices, that directly shape plant phenology and anatomy, and exert selective forces that in the long run will determine heritable differences between populations [6–9]. These can be uncovered by growing populations from diverse environments in a “common garden” experiment (e.g., [10]). Being part of the Fertile Crescent, our region is the cradle where many crops such as durum wheat, barley, pea, lentil have been domesticated [11]. They continue to evolve under domestication to present days, and many wild relatives being part of a crop’s gene pool still grow in nature.

*Cucumis melo* L. is perhaps the most genetically diverse crop among the *Cucurbitaceae*. In the Western world, sweet dessert melons are best known. They include *Cucumis melo var. cantalupensis*, having climacteric, aromatic fruits, and *C. melo var. inodorus*, with non climacteric, long keeping fruits, but the species also includes wild accessions (*C. melo var. agrestis*) and a few non-sweet cultivated varieties ([12] and refs. therein). Among these, *Cucumis melo var. flexuosus*, the snake melon, probably represents an early stage in melon domestication, where fruits have become non-bitter, but not yet sweet, offering a refreshing vegetable that can be eaten fresh, pickled or cooked at the immature fruit stage, like squash and cucumber ([13] and refs therein; [14, 15]). Later towards maturation they become inedible. Plants of snake melon are monoecious, ovaries are pubescent, and the fruits, as well as the stems, petioles and flower pedicel are elongated. The Arabic name for the local snake melon, Faqqous, originated from the Hebrew term “Piquous” (of Greek origin), indicating removal of fruit soft hair before consumption. Faqqous was known to ancient Hebrews as Qishut, mentioned in the bible as a vegetable eaten in ancient Egypt, and in the Talmud as a common cucurbit vegetable, whereas the cucumber (*Cucumis sativus*) arrived to the region much later, and Today’s squash is of course an acquisition from the New World ([13] and refs therein). This implies that Faqqous had a very long, probably uninterrupted history of cultivation in our region, rendering it an interesting germplasm resource, whose genetic composition could reflect its ancient and local origins.

At present, snake melon represents a traditional vegetable crop, mainly grown by Israeli Arab farmers in the Lower Galilee and in the Palestinian villages of Judea, Samaria and Jordan Valley under rain-fed conditions. In the framework of an Israeli-Palestinian collaboration, a collection of Faqqous accession was assembled [16]. In the present study, samples of the collection were sown in a common plot in Israel, to attain a full description of their phonological and horticultural diversity. Such analysis could suggest factors and traits that could limit economic productivity and could be improved by future breeding. We also probed the genotypic diversity of the collection and assessed population structure, using the seq-DArT method [17, 18]. The data collected were correlated with climatic and geographical parameters, providing insight on the possible forces that have shaped snake melon landraces in this small but climatically diverse region.

## Methods

### Set up of “common garden” experiment

To set up the collection, a single open-pollinated fruit from approximately 10 individual plants of a given agricultural field was sampled and its seeds represented an accession. Accessions from the same field were evenly mixed and represented a population.

**Fig. 1:**
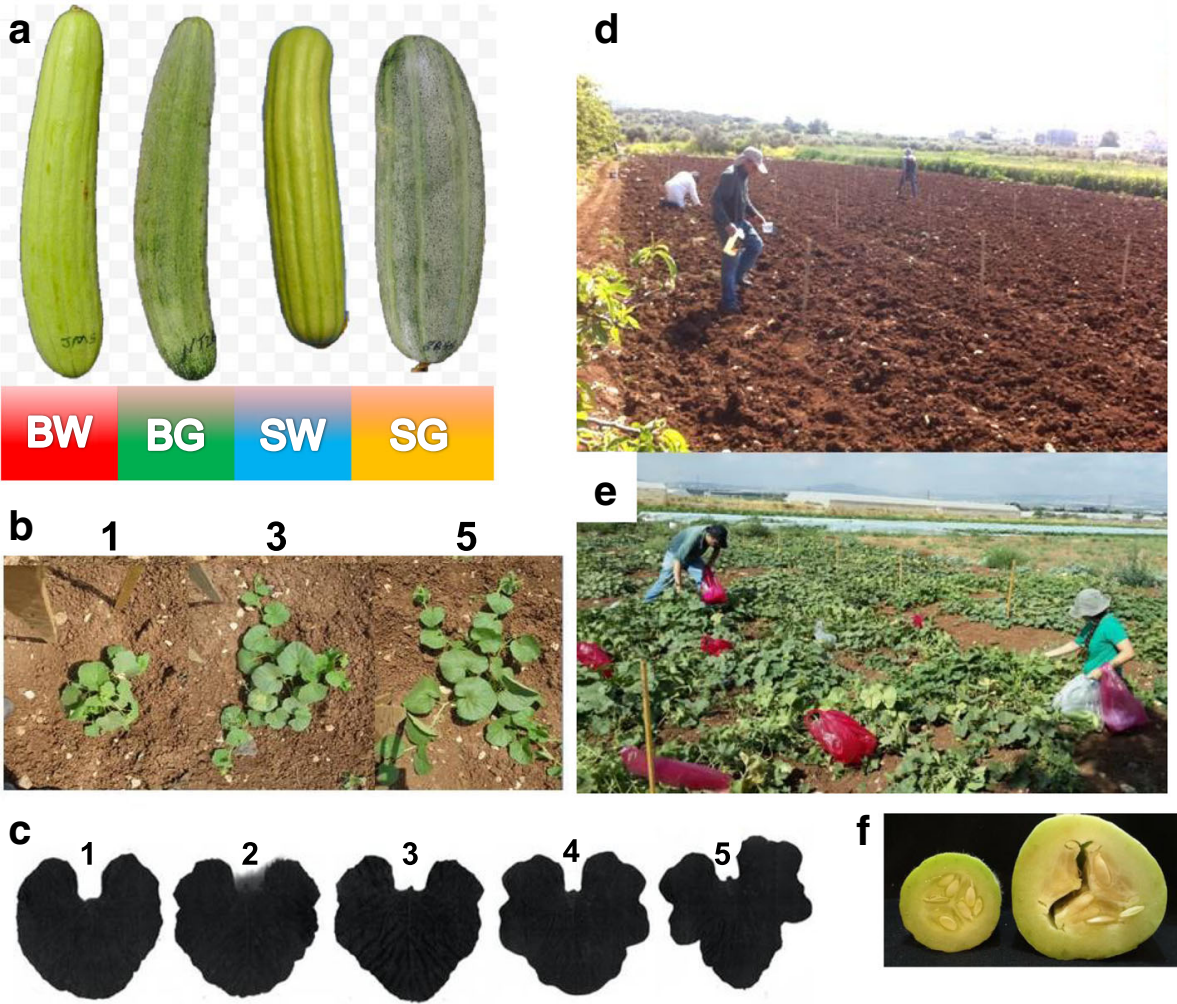
Faqqous types and phenotypic traits. **a** Four horticultural types of Snake melon grown in Israel and West Bank; (BW – Baladi White, SW – Sahouri White, BG – Baladi Green, SG – Sahouri Green). **b** Early vigor scores, visually scored on a 1–5 scale (vigor scores 1, 3 and 5 are shown). **c** Leaf shapes. **d** non-hollow (left) and hollow fruit (right), developing a space between placentas. **e** Direct sowing of common garden experiment in Sandala, Israel. **f** Experimental fruit harvest from each individual plant

The common Garden experiment was carried out in open field at Sandala, Yezre’el valley. We selected 43 populations, well distributed among geographical locations and Faqqous horticultural types (Fig. 1a). Each population included seeds equally sampled from all accessions. In addition, two commercial Faqqous varieties and two outgroups, FLI, a snake melon from India and FLT from Italy, were included. We planted the 47 populations × 12 plants per population, randomly arranged in three replicates (4 plants per replicate). The field was cleaned, plowed and pre-fertilized. Rows were spaced 2 m and plants within rows were 1 m apart. We sowed on 23rd March 2015 4–5 seeds at each position, watered each sowing spot by hand with ~ 250 ml and again after 5 days to assist establishment, and did not provide any further irrigation. During April development was slow due to the cold spring of 2015. Young plants were thinned to single plant/position. Harvest started at ~ 55 d post sowing, continued every 3–4 days, and lasted till late June, when the field started to decline, possibly due to water deficit and disease development. Field was regularly monitored for diseases and pests. Samples of the collection are available via the Israeli Plant Gene bank (samples No. 300031–300089), and the full data analyzed in this study is linked to each of these specimens via the gene banbk Catalogue and can be accessed by clicking “view information” and “attached files”.

### Phenotypic scoring

Morphological, phenological and horticultural traits were scored individually for each plant.

*Early Vigor (coverage)* of plants at the vegetative phase was evaluated 48 days after sowing. Each plant was scored twice by independent persons. We performed a qualitative visual score, where 1 represented the smallest plants and 5 – vigorous, largest plant (Fig. 1b). 2). We also counted all branches that exceeded 20 cm length and recorded the length of longest branch, as additional measures of early vegetative vigor. Since these measures were positively correlated we further analyzed only the visual scores.

*Female flowering*. We scored the earliest presence of open female flowers at three dates, starting 54 d post sowing, to attain good estimation of female-earliness. We also counted the female flowers aged from 2 days before anthesis to two days post anthesis in a continuous 11 day window in the beginning of female flowering, as a quantitative estimate of the total number of female flowers that develop on each plant.

*Leaf morphology*. Leaves were sorted in 5 different shapes (Fig. 1c), distinguishable by the lobes of varying depth.

*Yield measurement and fruit traits*. Immature fruit of snake melon are harvested continuously, every 2–3 days. We performed five “sample harvests” to provide yield estimate. Between these “snapshot harvests” the farmer made additional commercial harvests that were not recorded. Fruit longer than 10 cm were picked, and their number and total weight were recorded for each plant.

*Fruit morphology and quality*. In the two of the experimental harvests, the fruit from each plant were described. Two fruit from each plant (the largest and the smallest) were analyzed. Morphological traits were recorded as follows:

*Color*. 1 - light green, 2 - medium green, 3 - dark green, 4 - marbled (finely dotted over a gray background). *Dots:* present or absent. *Straightness*: 1 - straight, 2 - bent, 3 - winding. *Fruit shape* was determined as the length to diameter ratio that remains rather constant along fruit development. Diameter was measured in the middle of the fruit. *Ribs/furrowing* of the rind: 1 - deep, Sahouri like (few well demarcated, parallel furrows); 2 - like 1, but shallow; 3 - Baladi like (dense, less ordered wrinkles), deep furrows; 4 - like 3, but shallow (Figs. 1a, and 3).

For taste and texture evaluation, at each harvest two fruit each plant were assayed by a team of three participants. *Bitterness*: present/absent. *Taste*: 1 - tasty, 3 - not tasty. *Texture:* 1 - hard and crispy, 3 - soft. *Hollow fruit* (space between pericarp and placenta): present/absent (Fig. 1d).

*Late vigor*. Later in the season (73 d - 94 d after sowing) we scored six times the general vigor of the plant, until the field finally collapsed in July. We used a visual scale from 0 – dead plant to 5 – green and vigorous, the area under the plot providing an integrated, inverse measure of the senescence process.

### Genotyping by diversity Array technology (seqDArT)

Young leaves or side shoot tips were collected for DNA extraction from individual plants of two of the replicates, refrigerated in the field and stored frozen in the deep freezer. A total of 94 DNA samples from 23 populations × 4 individuals and two outgroup samples were prepared by the CTAB method [19], and sent for seqDArT genotyping at DArT, Canberra http://www.diversityarrays.com/dart). Genotyping protocols were as detailed by Al-Beyroutiova et al. [20], except for replacing HpaII with MseI digestion. Amplicons containing SNP were located on the melon genome [21]. Genotyping data was analyzed using the GenAlEx 6.502 software [22].

### Determination of soil type, soil properties and climatic data

Soil samples were collected from each collection site and visually inspected. Soil type was determined according to the detailed soils map of Israel based soil surveys by the Agricultural Research Organization and Soil Conservation and Drainage Department (http://www.govmap.gov.il; [23]. Calcium carbonate content of the soil was determined according to Rowell [24] using a devise modified by M. Sharabani, Geography and Environment Dept., Bar Ilan University. The % hygroscopic water content was determined by weighing the air-dried soil (Sw), then oven-drying for 24 h at 105 °C (Sd), calculated as 100 × (Sw-Sd)/Sd).

Climatic data based on yearly averages between 1960 and 1990 were taken from http://www.bioclime.org. Annual evaporation data was received from Dr. Noam Halfon, Israel Service of Meteorology.

### Statistical analysis

#### Quantitative traits

The following traits were statistically analyzed as specified below: taste, texture and proportion of hollow fruits; early, late and total yield; early and late vigor; number of female flowers and days to first female flower; fruit taste, texture and hollow fruit. In order to select the proper statistical tests for each trait, we checked the Normality of distribution of the traits using the Kolmogorov-Smirnov test on the ~ 564 individual plants in the experimental field. Traits exhibiting significant deviation from normality were transformed using logarithmic and square-root transformation, respectively, and tested for normality. In case transformation resulted in normal distribution, we used the transformed data for further testing.

For traits with normal distribution, parametric tests were used: Pearson correlation, Tuckey test, 1-way ANOVA followed by Tuckey post hoc test for differences among groups). For traits with non-Gaussian distribution, non-parametric tests were used: Spearman correlation, Kruskal-Wallis rank sum test for differences among groups, followed by Mann Whitney pairwise comparisons, corrected for multiple comparisons. The tests were run using R (The R core development team; https://www.r-project.org/) and SPSS (IBM SPSS Statistics - Essentials for Python 23.0, SAS Institute, Cary NC).

All the above ten traits had a significant deviation from normality (not shown), and logarithmic transformation did not improve the distribution. Upon square-root transformation (and block-correction, see below), Early Yield, Late Yield, Total Yield and Female-Flower Number attained normal distribution (*p* = 0.83, 0.12, 0.43, 0.34, respectively) in the Kolmogorov-Smirnov test, while the distribution of the remaining five traits remained non-normal (*p* < 0.05).

#### Correction of block effect

We checked whether the three replicates (blocks), located along a moderate slope, could differ in plant performance. We used 1-way ANOVA for early and total yield (after sqrt transformation), and Kruskal-Wallis test for the other five traits. Six traits showed a significant block-effect (*p* < 0.05), and the seventh trait, late vigor (“senescence”), was near-significant (*p* = 0.08). To compensate for such effect we performed a correction, adding/subtracting the difference of the block average from the whole field average; the remainder of the analysis was carried out using block-corrected values.

#### Dendrograms of accessions

A dendrogram based on morphological traits was plotted using the Hierarchical cluster analysis, “between group linkage” method. Distance values were computed by dissimilarity squared Euclidian distances, rescaled between 0 and 1 (SPSS, IBM SPSS Statistics - Essentials for Python 23.0, SAS Institute, Cary NC). A Neighbor Joining tree was inferred from the populations SNP allele frequencies using the DIST.GENPOP function in ADEGENET R package, to calculate Nei genetic distances and NJ function in APE R package.

#### Correlation between climatic data and plant data

The Random forest algorithm implemented in the Boruta R package was used to explore the most important variables of climatic data that are correlated with quantitative morphological traits, coefficients of variation (CV) of the traits, and molecular diversity. All climatic variables were used in one regression model. Spearman test for non-parametric correlation was used to estimate the goodness of fit (r) and the direction of the correlation.

## Results

### Evaluation of a snake melon collection in a common garden set up

**Fig. 2:**
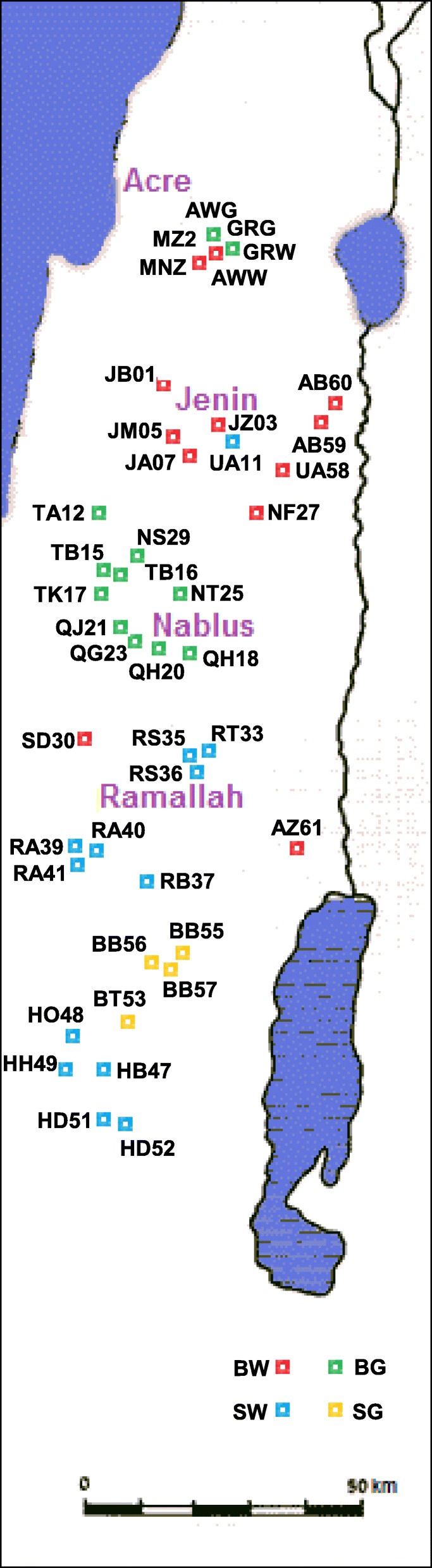
Collection site map. Sites are indicated by the population codes used in Table 1. Colors refer to four horticultural types (BW – Baladi White, SW – Sahouri White, BG – Baladi Green, SG – Sahouri Green

In a previous study, snake melon (“Faqqous”) accessions were collected in Israel and the West Bank [16]. Accessions included seeds from a single plant, resulting from open pollination in a farmer’s fields. Populations were assembled by mixing 10 random-selected accessions from the same farmer’s field. Forty-two such populations, or landraces, were selected for the present study, aimed to evaluate phenotypic and molecular diversity and investigate population structure in a common garden set-up, where accessions are grown side by side and compared. In addition we grew two exotic snake melon accessions, FLT from Italy and FLI from India, as well as two commercially available varieties from Israel: GRG and GRW, Agrodeal Ltd., Bet Natufa valley, and MRA, imported an unknown origin. Figure 2 shows the geographical origin of the populations, ranging from Arrabe in the Lower Galilee to the Hebron Area in South Judea. These sites differ in seasonal temperatures, annual precipitation and soil type. The populations belonged to four horticultural varieties: Baladi, which is long-fruited, densely wrinkled, further divided into light green (“White”) and dark-green (“Green”) forms, and Sahouri, with shorter fruits having straight, parallel furrows on the fruit skin, similarly divided into white and green forms (Fig. 1a). Table 1 summarizes the data regarding the different populations: collection site coordinates, horticultural variety, number of plants assayed in the experiment, soil type, soil parameters measured on samples from collection sites, main climatic parameters; a full list of climatic parameters is included in Additional file 1: Table S1.

**Table 1:**
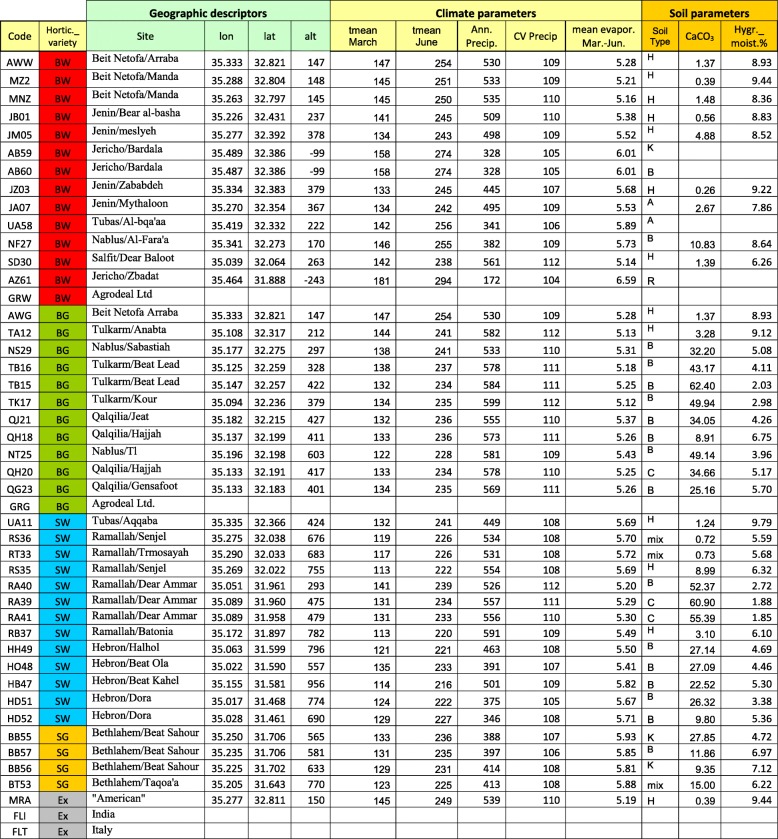
Data on landraces used in this study and their respective collection sites

Horticultural variety: BW, Baladi White, BG, Baladi Green, SW, Sahouri White, SG, Sahouri Green. Site: name of village/district. GPS coordinates: lon, longitude, lat, latitude, in decimal degree. Alt, altitude in meter. Ecogeographical variables, measured over three decades: tmean March, June: mean temperature in March and June, respectively. Ann. Precip, CV: annual precipitation in mm and its coefficient of variance. Mean evapor. March–June: mean evaporation in mm, March–June. Soil parameters: Soil type, A, Terra Rossa Soils. B, Mediterranean Brown Forest Soils. C, Rendzina Soils Of Mountains. H, Alluvial Soils. K, Rendzina Soils of Valleys. R, loessial serozemes, and mix, mixed soil. CaCO3: calcium carbonate content. Hygr. moist.%: percentage of hygroscopic moisture content

Twelve plants of each population, (3 replicates × 4 plants, ca. 560 plants in total), were sown in a common field in Sandala, Yezre’el Valley, following the traditional, rain-fed agricultural practice used for snake melon, as described in the Methods. Phenotypic analysis, conducted by scoring each individual plant, was divided into morphological traits, mostly scored as discrete states, fruit quality parameters of interest to consumers (measured semi-quantitatively), and horticultural traits measured quantitatively.

### Variation in morphological traits

The morphological-qualitative traits were as follows: fruit shape (L/W ratio), fruit color, presence of dots, rind furrows, fruit straightness, leaf shape. Scoring was done as detailed in the Methods section.

Additional file 1: Table S1 provides the scoring data regarding these traits. For each trait, it provides the segregation of the trait-states among individual plants.

**Fig. 3:**
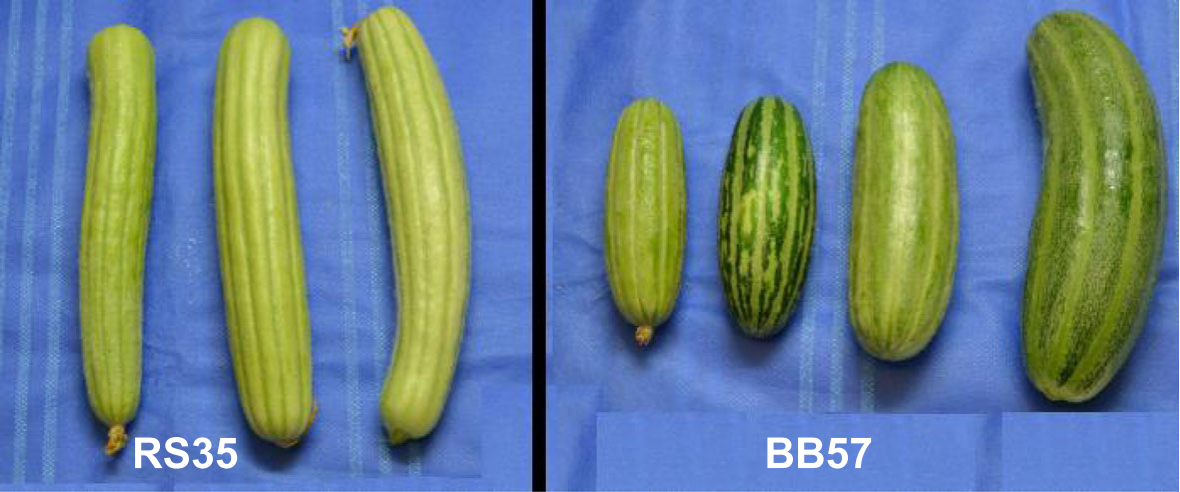
Populations differ in morphological variation. Representative fruits of a uniform Sahouri White population (RS35), compared to morphologically variable fruits of a Sahouri Green population (BB57)

We detected significant morphological variation in most of the populations. Some traits, such as skin color and leaf shape, showed high uniformity within populations, whereas groove morphology (furrowing) and fruit bending were more variable, perhaps because they vary during development, or because they are more genetically variable. Variability was prominent in certain populations, e.g.*,* Sahouri Green BB57, where dotted, darker and lighter fruits, with shallow / deep furrows were seen, while others such as White Sahouri RS35 looked uniform (Fig. 3).

Fruit dimensions constantly change due to rapid growth, but the length:width (l:w) ratios are rather constant from anthesis to ripening [25, 26], and provide a solid morphological character. When we plotted the l:w ratios from smaller and larger fruits in the same individual plant, we obtained a very high correlation (R^2^ = 0.97). The ratio (in addition to the furrowing pattern) readily distinguishes Sahouri (4.1 average ratio) from Baladi (5.3 ratio), while some exotic snake melons such as FLI have very elongated (15 ratio), snake-like fruits that are not found in our region. When we classified leaves by their shape (Fig. 1c), we noticed that the prominent leaf types were 3 and 4, with all Sahouri populations displaying state 4 and Baladi- state 3. FLI had Type 5 leaves, whereas leaf shapes 1 and 2 that are common in sweet melons, were not found consistently in our collection. Dotted fruits were quite rare, and bending was more common in Baladi than in Sahouri.

**Fig. 4:**
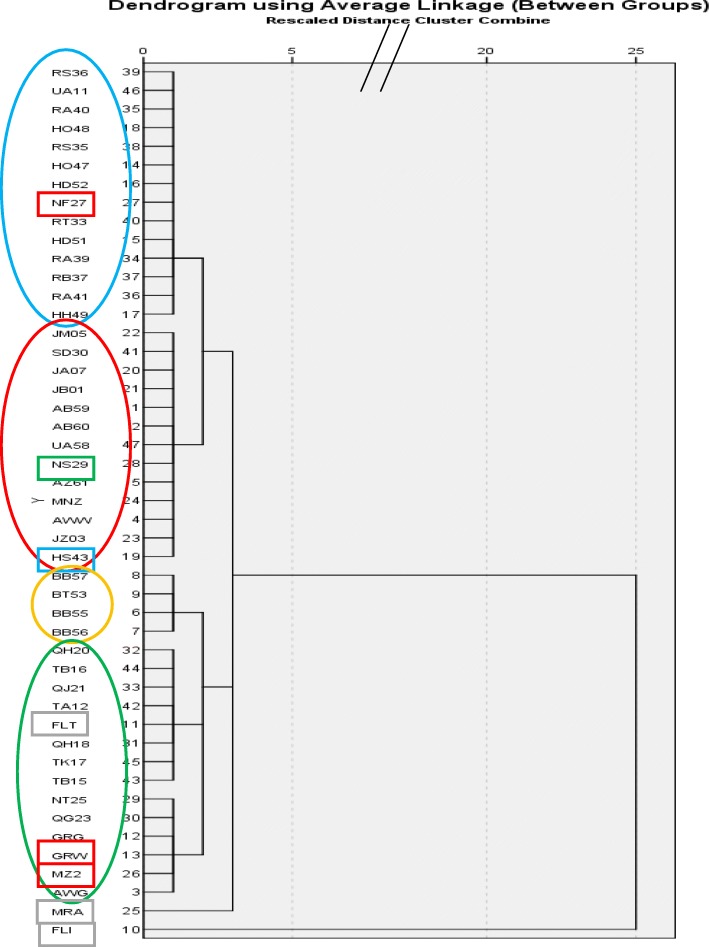
Dendrogram showing inter-population relationship based on six fruit and leaf morphology traits. Traits were: leaf shape, fruit straightness, fruit color, fruit skin dots, fruit ribs (grooves), fruit length/width ratio. Major clusters represent horticultural varieties with few exceptions. Clustering was performed using Hierarchical cluster analysis (SPSS; see Methods)

We depicted the relationship among populations based on morphology. For this purpose we calculated from the six qualitative traits an Euclidean squared dissimilarity matrix between all possible pairs of population. Based on the latter matrix, a dendrogram was plotted using Hierarchical cluster analysis (Fig. 4). The dendrogram differentiates our accessions into 6 main clusters, four of them separate the four horticultural types with few exceptions, while two separate branches lead to FLI (Indian snake melon) and MRA (an imported Faqqous variety).

### Fruit quality evaluation

Four fruit quality traits, namely the presence of bitterness, general taste (of non-bitter fruits), presence of a hollow fruit and fruit texture were evaluated and scored as described in the Methods section. Snake melon is eaten immature about one week post anthesis, and mature fruits become inedible. We tasted, in replicated trials, smaller and larger fruits of each plant (at 6–7 dpa and 8–9 dpa, respectively), the younger ones are preferred by consumers while the larger stage is still acceptable.

Bitterness was uncommon and few bitter fruits were found in 11 of the populations (mostly a single plant/fruit out of ~ 12), representing all four horticultural varieties. Populations AB60 from Bardala (N. Jordan Valley), QG23 (Qalqilia area) BB56 (Bet Lehem) and HS43 (Hebron area) stood out, with ~ 25% of the plants bearing bitter fruits.

**Fig. 5:**
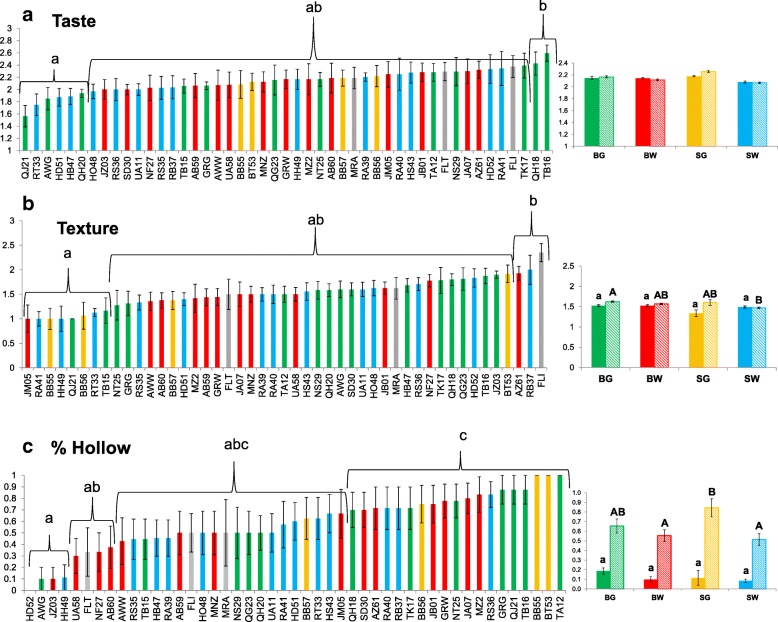
Ranking of landraces for fruit quality parameters. Fruits picked at 6–7 dpa (small) and 8–9 dpa (larger) were assayed. Left: average and SE of each population (smaller fruit data is shown for taste and texture, larger fruit data for hollow proportion). Right: average and SE of each variety, separately for small fruits (filled columns) and larger (hatched columns) fruits. **a** – Taste: 1- tasty, 3- insipid. **b** – Texture: 1 – crispy, 3 – soft. **c** – Frequency of hollow fruit: 1 – present in all plants, 0 - absent. Four horticultural varieties are marked by color as in Fig. 1. Letters depict significantly different values, according to 1-way Kruskal-Wallis followed by Mann-Whitney test

Figure 5 depicts the average scores and SE of taste, texture and presence of hollow fruits in all 47 populations and among the four horticultural varieties. There was a rather wide range of taste scores (Fig. 5a), from 1.5 (most tasty) to 2.6 (insipid). However, having included 47 populations in the ANOVA and post-hoc tests makes statistical threshold very stringent and only the extreme values attain statistical significance. The populations that attained the best scores belonged to the Sahouri White (SW) and Baladi Green (BG) horticultural types. Variation among populations from the same horticultural varieties was large, for example, different BG populations scored among the best and the worst tasting. The larger fruits did not differ on average from the younger ones, probably because the larger fruits were also relatively young. There was variation in texture as well, without clear demarcation between horticultural types and the two picking stages (Fig. 5b). A crispy texture is preferred by the consumer and considered more palatable.

Separation of the mesocarp from the placenta (Fig. 1d) is frequent in mature fruit, but is less desirable in immature fruit at the commercial stage. The proportion of hollow fruit among the smaller fruit of the four varieties ranged between 9 and 17%, and quickly increased to 44–64% in the larger fruit with higher ratios in SG landraces (Fig. 5c). Among the individual populations, BB55 (SG), HH49 (BW), JZ03 (BW) had the smallest proportions of hollow fruit.

**Fig. 6:**
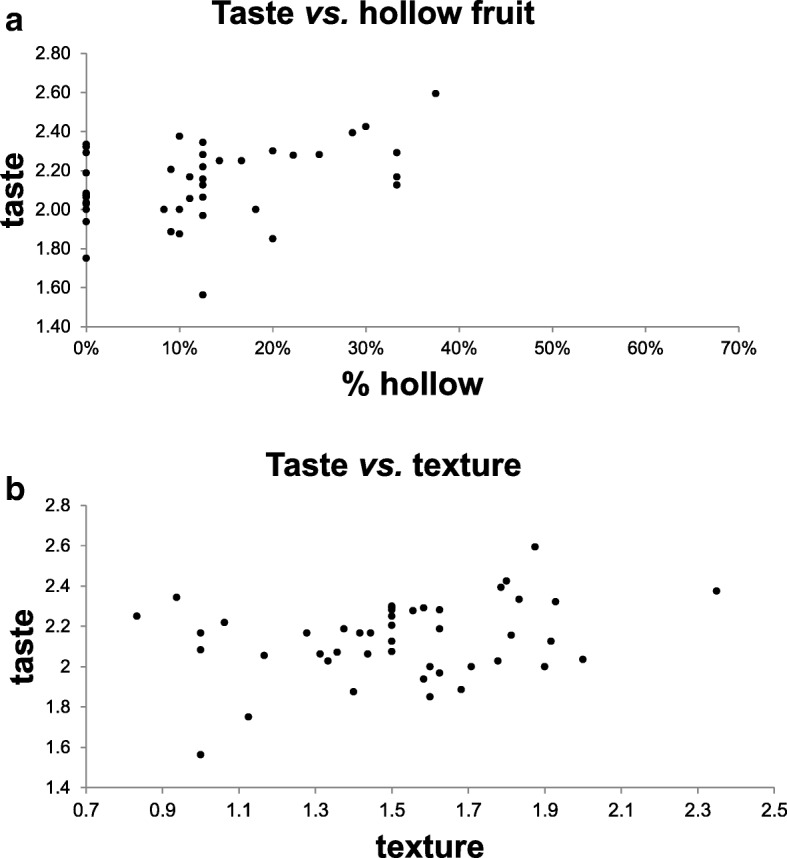
Scatter plot showing the relationship between average fruit taste and other fruit quality traits, scored on 47 populations. **a** - Relationship between taste (relative scores: 1- tasty, 3- bland) and texture (relative scores: 1-crispy, 3-soft). **b** - Relationship between taste (relative scores: 1- tasty, 3- bland) and frequency of hollow fruits (1 - hollow, 0 - no space between mesocarp and placental region)

In an attempt to develop selective criteria for improving fruit quality, we asked whether there is a correlation between our score for “general taste” and the other two traits, hollow fruit and texture. Figure 6 plots the relationships. There is no linear correlation between taste and either trait. Still, the scatter plot having relatively few data points in the bottom-right quadrants suggests that crispy and non-hollow fruits are not necessarily more tasty, but softer and hollow fruits are seldom associated with very good taste. This suggests that the three quality criteria often referred to by consumers are related but do not overlap, and selection against soft and hollow fruits (as well as bitter fruits) is required but cannot replace selection for an overall good taste. The ability to maintain a crispy texture devoid of spaces for a longer period would preserve fruit quality for a few more days and could enable, to some extent, less frequent picking of larger fruits.

### Quantitative phenological/horticultural traits

The most important trait for future improvement of Faqqous is yield, a complex trait that is probably affected by numerous environmental and plant developmental factors. We compared the average values and extent of variation within the 47 “populations” and four horticultural varieties, for seven selected traits related to plant performance in general and fruit yield in particular. These included early plant vigor, the number of days until the appearance of the first female flower, number of female flowers in a determined time window, early, late and total fruit yield, and late vigor, measured towards the end of the season (an inverse measure of senescence).

**Fig. 7:**
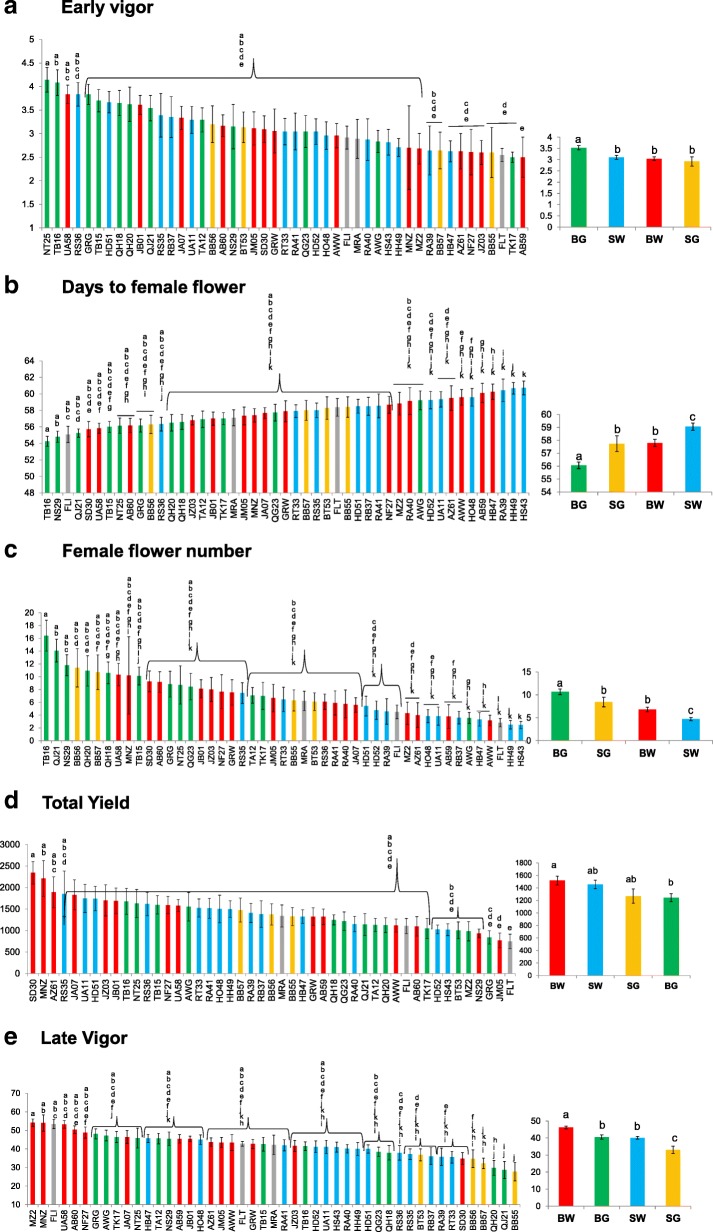
Ranking of 47 populations for quantitative traits, measured as detailed in the Methods section. Left: average and SE of each population, right: average and SE of each variety. Letters depict statistically significant differences values (see Methods section). **a** – Early vigor. **b** – Number of days from sowing to the opening of the first female flower. **c** – Number of female flowers per plant in an 11 days window. **d** – Total Yield of marketable fruits picked during five experimental harvests. **e** Late-age vigor of plants, integrated from a series of visual-scale observations. Four horticultural varieties are marked by color as in Fig. 1

The earliest trait that could affect plant establishment, biomass and yield is the early vigor, manifested by initial growth rate and soil coverage. It was estimated 48 days post sowing, and population average values ranged between 2.5 and 4.2 (Fig. 7a), indicating large variation in this trait. The most vigorous or early-establishing landraces were three Baladi samples, NT25 from Tell, Nablus, TB16 from Beat Lead, Tulkarm, and UA58 from Al-bqa’a, Tubas. Among the four varieties, BG stands out for higher vigor.

Male flowers reached anthesis six weeks after germination, and one week later the female flowers, that are fewer and borne singly on side branches, first appeared. Plants that exhibited fast vegetative growth have the potential to attain female flowering earlier, and those having a stronger or longer female phase could have a larger yield. The number of days from sowing to first female flower ranged between 54 and 61; among the four varieties BG was earliest and SW the latest (Fig. 7b). However, variation within populations was large and individual plants could differ by 4 days. When we compare the number of female flowers that opened in an 11 day window, large variation was apparent, ranging between two flowers in SW population HS43 from Suba, Hebron and 16 flowers in BG population TB16 from Tell (Fig. 7c). Among varieties, BG displayed more profuse flowering and SW had the fewest female flowers. Because the time window selected was at the onset of flowering, an inverse correlation between the two femaleness parameters was expected; measuring an additional time window later in season would have been desirable but would interfere with fruit picking and yield measurement.

Picking of marketable fruit (10–30 cm long) was done every 3 days, starting after 55 days, and lasted for ~ 40 days. Because of the indeterminate growth habit and continuous requirement to harvest immature fruit (non-harvested fruit would inhibit further fruit set), it was not technically possible to measure the actual yield of each plant. Instead, we performed five “snapshots” or sample-harvests, in which fruits of individual plants were picked, counted and weighed, and, in-between, the farmer performed additional harvests and “cleaned” the plants. Total Yield is the sum of all five experimental harvests, and the actual yield along the season can be estimated by multiplying the total experimental yield by 2. The average experimental yield in our experiment was 1.4 kg/plant (representing a full yield estimate of 2.8 kg) and ranged between 0.8 and 2.3 kg/plants. Figure 7d presents the total yield (summed over the five experimental harvests) of 47 populations and four varieties. The highest yielding populations were SD30 from Dear Baloot and MNZ from Kafr Manda, and the lowest yielding were FLT from Italy and JM05 from Meslyeh. Among the varieties, BW ranked significantly higher and BG – lower.

Later in the season, we scored plant vigor using a qualitative scale (see Methods section), and integrated observations taken on 6 different dates (6 June - 5 July) into an “area under survival curve” value, where higher values represent higher late-vigor. Fig. 7e plots such values for each population and for the four Faqqous varieties. Late vigor scores varied from 27 to 54 units, Green Sahouri senesced earlier than the other varieties, while White Balladi exhibited, on average, prolonged vegetation.

#### Correlations among traits

Correlation (or lack of correlation) between quantitative traits could provide useful insight on factors that determine such traits, and generate ideas regarding future genetic improvement, based on the large phenotypic variation apparent in our collection. Spearman correlations were computed between all pairs of seven traits, using the individual scores from ca. 500 plants.

**Fig. 8:**
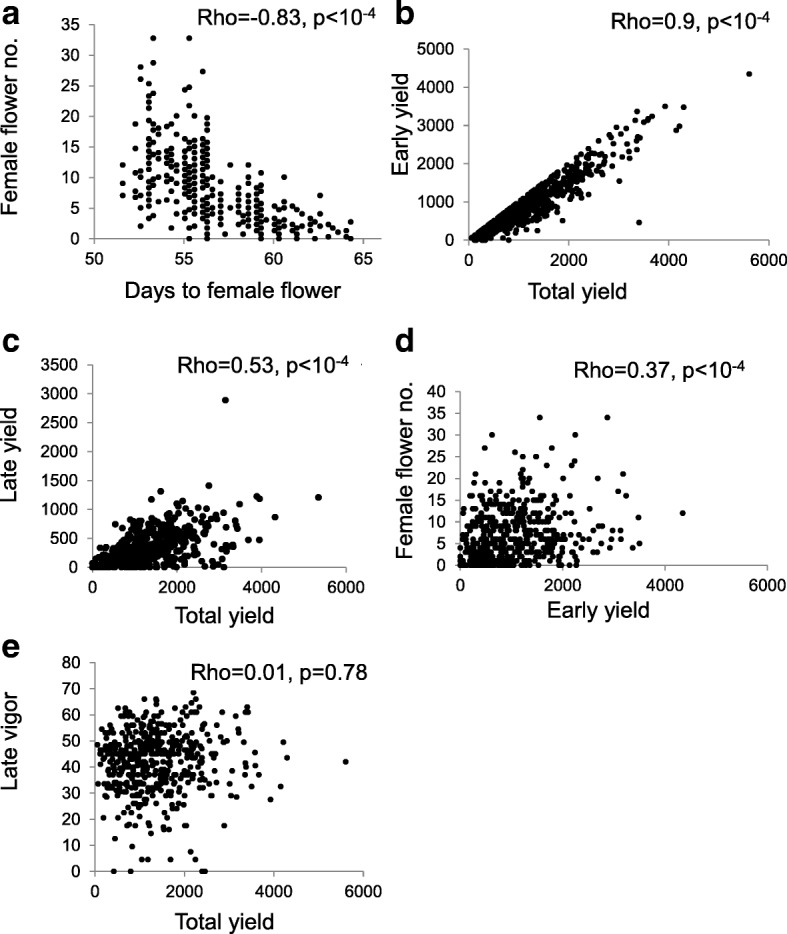
Correlations between selected pairs of quantitative traits, measured in ~ 560 individual plants. Spearman correlation coefficients (Rho) and their statistical significance are shown. **a** – Correlation between female flower number and female flowering date; **b** – Early yield and total yield; **c** – Late yield and total yield; **d** – Female flower number and early yield; **e** – Late vigor and total yield

**Fig. 9:**
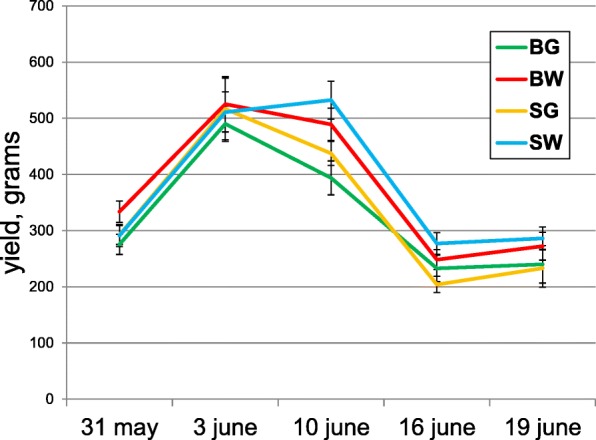
Fruit yield per plant, harvested at five dates in May and June. Yields are shown separately for each horticultural variety. Four horticultural varieties are marked by color as in Fig. 1

Not surprisingly, the two parameters measuring the degree of femaleness, days to first female flower and number of female flowers over an 11 day window are strongly and inversely correlated (Spearman coefficient = − 0.83, *p* < 10^-4^; Fig. 8a). Early Yield (the first three harvests) was strongly correlated with Total Yield (all five harvests, Spearman coefficient = 0.92, *p* < 10^-4^; Fig. 8b). The correlation of Late Yield (last two harvests; Fig. 8c) with the total yield is smaller but significant (0.53, *p* < 10^-4^), suggesting that the first harvests have a larger contribution to total yield. This is also apparent by plotting the average yield of each variety at each harvest (Fig. 9), showing that the yields started to decrease towards the third harvest and remained low at the 4th and 5th harvest. The correlation between early and late yield was much smaller (0.165, *p* = 0.002), suggesting that populations can be productive either earlier or late in the season, but also, that there is no necessary contrast or compensation between the early and late phases of production; populations exist that produce well both early and late (e.g.*,* population MNZ from Kafr Manda and SD30 from Dear Baloot), and these could be selected for varietal improvement.

Early vigor was strongly correlated (0.65, *p* < 10^-4^) with the number of female flowers that was measured, as explained above, in an early time window, and with the days to first female flower (− 0.67, *p* < 10^-4^). Female flower number was correlated with early yield (0.37, *p* < 10^-^^4^; Fig. 8d). The correlations of early vigor with early and total yield had smaller, but statistically significant, correlation coefficients (0.28, *p* = 0.0004 and 0.27, *p* = 0.001, respectively). This suggests that while early vigor and establishment contribute to yield, plants can later catch up and compensate for this parameter. On the other hand, we saw no correlation between late vigor*,* i.e., whether a plant remained green longer, and its total yield, or even the late yield (Fig. 8e). It could be that the late-senescing plant produced a low yield in the earlier (or even later) phases, such that late senescence did not correlate with higher productivity. The lack of negative correlations could suggest that variation in both traits (early vigor and late vigor, and possibly prolonged productivity) could be combined by breeding.

### Genetic diversity revealed by seqDArT

Seq-DArT (Diversity Array Technology [17], is a high-throughput genotyping method that generates sequence-based markers. These include co-dominant single nucleotide polymorphism (SNP), as well as dominant markers representing presence/absence of amplicons (“in silico DArTs”). In order to obtain a broad picture of the genetic structure in our snake melon collection, we selected a subset of 23 populations that represent the four horticultural varieties and the different geographical regions, and sampled four individual plants/population, in addition to two individuals of the out-group varieties, FLT (a Faqqous inbred genotype from Italy) and Vedrantais (a sweet melon inbred cultivar).

A total of 4324 SNPs have been generated, along with 4162 “in-silico DArT” dominant markers. We used the SNPs for further analyses, removed the lower quality data, and were left with a set of 2748 SNP with complete data for all individuals and significant frequency of both alleles. In total, 2460 of the SNP could be mapped to the sequenced melon genome, and 288 remained unmapped. The number of mapped SNP per chromosome ranged between 251 in chromosome I and 140 in chromosome X (Table 2). The average DArT marker distribution is 1 SNP marker/136 kb of the 375 Mb genome [21], and according to the linkage map of Perin et al. [27], 2 SNP per centiMorgan.

**Table 2:** Distribution of DArT markers on the melon genome. Chromosome length in cM is according to Perin et al. [27]

chromosome	Map Length (cM)	SNP	silicoDArTs
I	103	251	348
II	98	222	257
III	154	187	297
IV	170	238	317
V	97	191	280
VI	100	247	340
VII	154	204	240
VIII	156	229	281
IX	111	192	207
X	76	140	179
XI	104	164	199
XII	88	195	275
not mapped		288	942
total	1411	2748	4162

Based on DArT genotyping, we asked how much molecular variation is present in the collection, and how is it partitioned *within* individuals (in the form of heterozygosity), *between* individuals in a population, and among populations. Is heterozygosity higher or lower than expected, and what can be learned from this on population structure and reproductive strategy/ history of Fakus in our region? Does the molecular data divide the collection according to population, horticultural variety, geographic region?

Using the GenAlex software [22] we applied the Frequency and HFP (Heterozygosity, F-statistics, polymorphism) functions calculated for each of the 23 populations and for the entire collection (92 samples). In each population, the percentage of polymorphic loci among the four individuals was high and ranged between 72 and 46% (Table 3). The total unbiased heterozygosity expected (uHe), summed over all loci and corrected to account for small population size (a widely accepted measure of genetic diversity in populations) ranged between 0.46 in population TB16, a Green Baladi landrace from Beat Lead, and 0.32 in MZ2, a White Baladi from Kafr-Manda. In the two inbred samples, only 4% of the loci were heterozygous in Védrantais, and 1% in FLT.

**Table 3:** Statistics of genetic variation 23 melon populations as measured by 2748 high quality SNP loci, GenAlex software

Pop	N	P	I	Ho	He	uHe	F
SD30	4	65.8%	0.545	0.408	0.372	0.425	− 0.105
JA07	4	62.6%	0.516	0.394	0.353	0.404	−0.118
NF27	4	697%	0.564	0.433	0.388	0.443	−0.116
JM05	4	60.9%	0.491	0.405	0.333	0.380	−0.215
MZ2	3	46.1%	0.386	0.189	0.265	0.319	0.245
AB59	4	66.2%	0.534	0.322	0.366	0.418	0.083
AZ61	4	64.1%	0.501	0.384	0.340	0.388	−0.137
UA58	4	66.0%	0.548	0.348	0.376	0.430	0.050
TB16	4	71.9%	0.576	0.438	0.398	0.455	−0.098
NS29	4	68.3%	0.550	0.429	0.377	0.431	−0.144
AWG	4	62.7%	0.497	0.407	0.337	0.385	−0.201
NT25	4	67.6%	0.573	0.524	0.395	0.451	−0.322
TK17	4	66.4%	0.521	0.320	0.355	0.406	0.077
TA12	4	70.0%	0.575	0.434	0.396	0.453	−0.105
UA11	4	53.1%	0.454	0.300	0.306	0.350	−0.024
HD51	4	66.9%	0.563	0.416	0.388	0.444	−0.077
RS35	4	70.0%	0.569	0.421	0.391	0.447	−0.081
RA40	4	66.9%	0.564	0.503	0.389	0.445	−0.275
RB37	4	69.3%	0.563	0.429	0.387	0.442	−0.112
HH49	4	65.5%	0.548	0.393	0.375	0.428	−0.062
BB57	4	66.7%	0.568	0.419	0.391	0.447	−0.077
BB56	4	68.0%	0.568	0.376	0.390	0.446	0.022
BT53	4	68.4%	0.555	0.367	0.380	0.435	0.019
Mean	3.720	65.4%	0.495	0.364	0.339	0.389	−0.084
SE	0.008	1.2	0.002	0.003	0.002	0.002	0.005

*N* number of individuals, *P* percent polymorphic loci, *I* Shannon’s Information Index, *Ho* Observed Heterozygosity, *He* Expected Heterozygosity, *uHe* unbiased expected heterozygosity, *F* fixation index

The Fixation index (“F”; Table 3) is calculated as 1-Ho/He. Most populations had a negative index, meaning that heterozygosity is higher than expected by Hardy-Weinberg equilibrium. Possible reasons for such situation are discussed below.

**Fig. 10:**
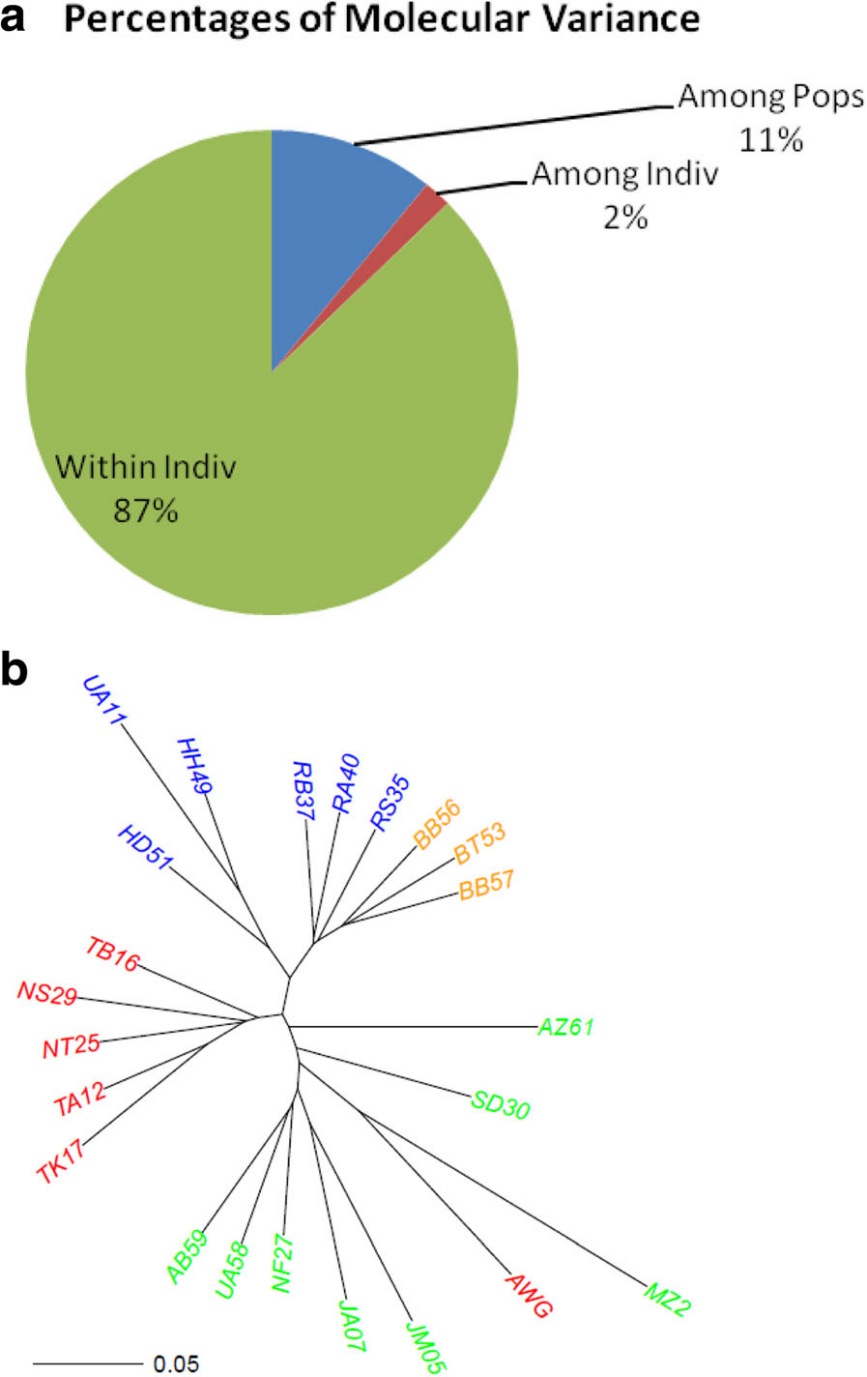
Population structure based on allelic frequencies of 2748 biallelic SNP loci among 23 populations. **a** AMOVA (Analysis of Molecular Variance) based on 2748 loci and 94 individuals from four horticultural types and 23 populations, using the GenAlex program. **b** Neighbor Joining tree inferred from the SNP allele frequencies using the DIST.GENPOP function in the ADEGENET R package. Nei genetic distances and NJ function were calculated by the APE R package. The four horticultural varieties are marked by color as in Fig. 1

AMOVA (Analysis of Molecular Variance) revealed a very high proportion (87%) of “within individuals” variation (i.e., heterozygosity), a small proportion of variation among different populations (11%), and a remaining very small proportion (2%) of variation among individuals within a population (Fig. 10a). Such structure could result from the high levels of heterozygosity, but also from frequent genetic mixing within and among populations. We depicted a Neighbor-Joining tree of the 23 populations based on the allelic frequencies of the 2748 SNP in each population (Fig. 10b). The large heterozygosity ratios compared to the smaller proportion of variation among populations revealed in the AMOVA plot are also reflected in the tree structure, where terminal branches were long compared to the short nodes separating groups of populations. Such “informative branches” are based on a small subset of SNP, and supported by low Bootstrap values (< 50), nevertheless they separated all four horticultural varieties very well: the two Baladi from the two Sahouri varieties, and within each main variety, into the green and the white variant, with a single exception, AWG.

**Fig. 11:**
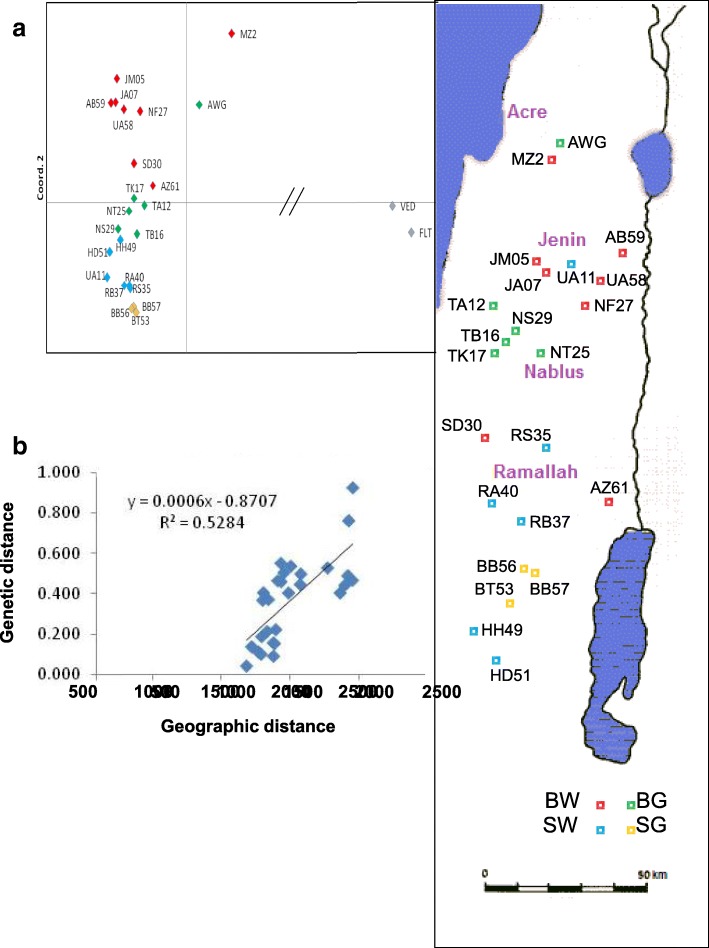
DNA profiles divide Faqqous varieties and reflect geographic separation. **a** Principal Coordinate Analysis (PCoA) calculated over all loci and 94 individuals from 25 populations, using the GenAlex program. **b** Correlation between genetic and geographic distances calculated by the Mantel Program from the 28 pair-wise distance values among eight White Sahouri populations

Principal Coordinate Analysis (PCoA) depicted in a more “objective” manner the overall relatedness among our samples (Fig. 11) and showed that the two “exotic” outgroups are very distant from local Faqqous. In addition, populations belonging to the same horticultural variety tend to cluster, indicating that the four varieties are, to some degree, genetically distinct. Moreover, the PCoA pattern is also related to the geographic map, suggesting that genetic relatedness is influenced by the geographic distance. We noted that one of the White Sahouri accessions, UA11 collected from Tubas (Jenin area), clustered with the other accessions from the Hebron area; in fact the farmer reported that the variety originated from Hebron. We used the Mantel test to calculate the correlation between geographic and genetic distances among the eight White Baladi populations that underwent DArT analysis, and are well spread geographically (the other varieties had less samples and narrower distribution). Indeed, a strong correlation was observed (R^2^ = 0.53, *p* = 0.01), suggesting that spatial separation between landraces underlies, at least in part, their genetic separation.

## Relation between geographic and climatic data and morphological/genetic variation

We asked whether landraces that evolved under domestication in different climatic regions responded to climatic variables in a recognizable way. We tested whether the geographic or climatic variables (geographic latitude and altitude; soil type and calcium carbonate content, and multiannual averages of evaporation and precipitation, minimum, average and maximum temperatures in the Faqqous growing season; Table 1 and Additional file 1: Table S1) are correlated with the seven quantitative traits measured, or with their coefficients of variation, or with the amount of genetic variation (unbiased heterozygosity, see above) of the snake melon populations. We ran a random forest regression analysis and detected seven statistically significant correlations (Table 4).

**Table 4:** Statistically significant correlations between population means, coefficient of variation (CV) of quantitative traits, or genetic variation, and long-term climatic variables, determined by random forest feature selection and Spearman correlation

Trait	Env. variable	Rho	*p* Value
TotalYield CV	tmax4	−0.4976	0.01567
Early TotalYield CV	tmax4	−0.5902	0.00302
Early TotalYield Mean	eva3	0.43873	0.03745
Vigor Mean	tmean3	−0.4917	0.01717
Female Fl. Date Mean	prec2	−0.4042	0.05572
Late Vigor Mean	tmin6	0.71803	0.00011
uHe	prec3	0.54437	0.00724

Tmax4, temperature maximum in April; eva3 – evaporation in March; tmean3, tmean6 – temperature minimum in March and June, respectively; prec2, prec3 – precipitation in February and March, respectively

The coefficients of variation of both the total yield and the early yield (two highly correlated traits) were negatively and strongly correlated (rho = − 0.5) with the maximum temperature in April. This parameter ranged between 30 °C in the Jordan valley and 22–23 °C in most regions. This could imply that extreme environmental conditions tend to reduce genetic variation, as shown by Peleg et al. [5].

More correlations were found between plant traits and climatic variables, but not soil or geographic parameters (Table 4). Early vigor was negatively correlated with the mean temperature in March, suggesting that cool springs could have selected quicker establishment of the plants. Early yield, on the other hand, positively correlated with evaporation in March. Early-season evaporation could reflect windy and/ or warm weather and this could favor early and higher yielding and perhaps a shorter life cycle. Late vigor, i.e., maintaining green foliage late in season, was very strongly correlated (Rho = 0.72) with minimum temperature in June. Such minima range between 14 °C in the Hebron area and 22 °C in the Jordan and Northern valleys. In the former areas, characterized by continental climate, plants could have evolved a shorter life cycle and senesced faster than in areas with warmer nights.

Finally, molecular variation, expressed as unbiased expected heterozygosity, was positively correlated with March precipitation, which range between 23 mm in the dry Jordan valley and 100 mm in Ramalla. In our Mediterranean, rain-limited area, less extreme conditions apparently favored genetic variation.

## Discussion

Snake melon (*Cucumis melo var. flexuosus*) is an ancient crop in Israel and Palestinian territories as well as other Mediterranean countries. In a previous study, a collection of landraces of Faqqous, the Palestinian snake melon, was assembled and a preliminary description provided based on observations in situ, at collection sites in Israel and the West Bank [16]. Faqqous is an important summer vegetable grown without irrigation in Arab villages, and has not been subjected to modern breeding practices [14, 28]. During the collection trips we learned that each farmer selects a few open-pollinated fruits to save seeds for the following season. We hypothesized that such germplasm harbors significant genetic variation generated by long-term evolution under domestication, and by exposure to the large variation in temperatures and precipitations found in this small but very diverse region. We also hypothesized that such variation could be further selected by future breeding programs, to increase Faqqous yields and tolerance to biotic and a-biotic stress.

Here we report the characterization of 42 landraces from Judea, Samaria, the Lower Galilee and Jordan Valley in a common garden experiment conducted in Sandala, Yezre’el Valley. Our purpose was to describe the collection in a comparative manner, assess its phenotypic variation and agronomic potential and derive genetic and agronomic insights for future conservation and breeding. Such an experiment complements in situ observations, where each variety is assessed in the site to which it has adapted; the common garden provides a uniform environment (sowing time, soil-water content, simultaneous assessment of parameters), that is essential to characterize the collection and select items from it. The same collection will need to be analyzed in additional environments to provide a full picture of genetic x environment effects on important traits.

*Cucumis melo* is a highly diverse species, traditionally divided in two subspecies: *ssp*. *agrestis*, with wild accessions having inedible, often bitter fruits, and *ssp*. *melo*, further divided to six varieties: *cantalupensis, inodorus, conomon, chito, dudaim* and *flexuosus* [12, 29]. The first two varieties consist of sweet dessert melons (climacteric and long-keeping, respectively), popular in the western world since the Roman period. The others are mostly non-sweet varieties, but in the Far East, parallel domestication gave rise to sweet cultivars of the Makuwa group. Stepansky et al. [12] used molecular markers on a 60-accessions core-collection that broadly supported such intraspecific classification, and placed snake melons closer to the sweet melon, in concordance with their Middle-Eastern origin.

It has been thought that *Cucumis melo* speciated in Africa, but this view has been recalled by Sebastian et al. [30], based on analysis of a diversified array of Asian and Australian species, from which both melon (2n = 24) and cucumber (2n = 14) must have originated. The first domestication site of melon is unclear, and multiple domestication events are likely. It has been suggested that melons were domesticated in two stages, first as a non-sweet vegetable whose fruit is eaten shortly after anthesis, and only later as a sweet fruit eaten mature. Faqqous and a few other melon varieties (e.g.*, conomon*) represent the first stage of domestication that required removal/mutation of bitterness genes. Cucurbit fruits produce the triterpenoid cucurbitacin, and interestingly such trait persists sporadically in the domesticated germplasm and needs to be selected against in crops such as cucumber and watermelon. Bitterness is affected by the environment, and bitter progeny may arise from crossing two non-bitter parents. In cucumber, it is controlled by several interacting genes [31, 32] that determine whether the foliage and/or the fruits are bitter. In our collection, 23% of the populations included bitter-fruited individuals, despite the obvious selection that had to be exerted against such deterrent trait. This is an obvious target for future improvement. The reasons for persistence of bitterness are presently unknown, perhaps poor heritability, or lack of awareness by growers who select the seeds. Growers’ folklore maintains that bitterness comes from pollen of nearby *Echballium elaterium*, a distantly related cucurbit weed.

A more realistic concern about Faqqous inter-crossing relates to the novel trend of consuming, as “Faqqous surrogate”, fruits of a sweet melon variety at the immature stage. As a result, Faqqous landraces are grown side by side with sweet melons and can cross via honeybees, resulting in genetic contamination of the traditional landraces. This further highlights the need for collecting and preserving the Faqqous germplasm.

Studies on melon diversity are numerous, but those focusing on snake melons are very few. Indian snake melons were described by Pandey et al. [28]; a Jordanian collection of 8 accessions was described by Abdel-Ghani & Mahadeen [33], who analyzed morphological traits and random amplified polymorphic DNA (RAPD) markers. Our study, with 46 populations × 12 plants/population provides a more in-depth evaluation of snake melons from a given area, with good genome sampling by a modern genotyping method.

When examining morphological traits in a qualitative manner, landraces from Israel/Palestinian territory fall into four main horticultural varieties: slender, wrinkled Baladi versus stout, straight-furrowed Sahouri, each of these including dark and light-colored accessions. Some populations appeared more variable that others and contained atypical fruits, indicating gene-mixing between populations. When examining fruit-quality traits*,* i.e., occurrence of bitterness, crispy texture, good flavor and hollow fruit, we observed significant variation within populations that could result from genetic variation, or from low heritability and difficulty in the objective assessment of such traits. Nevertheless, a few populations stood out as being better or worse than the majority (Fig. 5), and these should be rechecked in pre-breeding screens for stability of the trait. The correlation between good taste and crispiness or hollowness is not simple (Fig. 6), but hollow and softer fruits were often graded less tasty.

Yield improvement is an important breeding goal for 'Faqqous’ and progress towards such goal starts by evaluating diversity in yield, and in traits that could relate to yield. The randomized block experiment in a single environment allowed careful scoring of vegetative and reproductive growth of each individual plant. We detected significant variation in total yield (composed of early and late yields), in the early and late vigor representing, respectively, the establishment phase in young plants and the senescing phase at the end of the season. We also measured the degree of femaleness using two different parameters. Due to the high intra-population variability (later corroborated by our molecular data), differences among populations were statistically significant only between the populations from the two extremes of the spectrum (Fig. 7). Repeating such experiments with larger samples and fewer populations should confirm the heritability of such differences. In the present experiment, however, the large variation and statistical significance between extremes support the likely existence of useful genetic variation in the collection, both within and between populations.

We hypothesized that populations that can quickly establish a vegetative biomass and/or maintain a productive, green biomass later in the season (excelling in early and/or late vigor) would have higher yields. We also expected to find positive correlations between the femaleness parameters and yield, since in traditional varieties of cucurbit crops that have not been subject to modern breeding, female flowers are limiting, and represent an important yield component [34].

We plotted the Spearman correlations between quantitative traits, based on > 500 individual plant measurements (Fig. 8). Such plots indicated statistically significant correlations between early vigor, femaleness and yield. There was also large variation regarding performance late in the season, when some accessions stayed green for an extended period, when others had already collapsed. However, this potentially important trait was not statistically correlated with yield. This could suggest that different accessions employ different pathways/ adaptation strategies that could involve earlier or delayed fruiting, or even different ways to utilize soil water and resist stress. The large variation and lack of correlation between such traits actually indicate that exceptional genotypes are present in population that combine, for example, early vigor and late vigor, or high femaleness and high yield, and there is room for genetic amelioration by selecting superior trait-combinations.

Genotyping germplasm collections with markers can eliminate duplications and misnaming. More importantly, the distribution of diversity among accessions and collection sites can help understand population structure, and direct further collection efforts and germplasm maintenance (e.g.*,* [35–37]. Genotyping core collections using molecular markers has been facilitated by novel SNP discovery techniques, based on next generation sequencing methods (NGS; [38, 39]. These provide thorough genotyping with thousands of SNP per individual genotype, allowing association studies that locate important genes; as a result, genotyped core collections become a permanent, invaluable resource not only for conservation but also for genetic research. In this study, we used the seqDArT genotyping method that has been developed from a microarray-based platform into an NGS-based one [20]. We generated, in a cost-effective manner, 2460 best-quality SNPs, without missing points in the entire 94 sample-set. Over 90% of the SNP could be aligned, and were evenly distributed, on the melon genome; a larger number of dominant markers was derived as well.

Of the 2460 polymorphic markers, a very high proportion, 46–72%, were polymorphic within each population, considering the small sampling size (*n* = 4). AMOVA analysis and unbiased heterozygosity calculations show that heterozygosity is extremely high, 87% of the molecular variance being attributed to individual’s heterozygosity. This leaves small proportions of variation within and between populations (Fig. 10), and translates in higher-than-theoretically expected heterozygosity, and negative Fixation Indices, in 18 of 23 populations (Table 3). This is rather surprising, in view of the repetitive selection by the farmers who collect a very small number of fruits per field (~ 10, each with ~ 300 seeds, from a field of 1000 plants, to produce the 3000 seeds he may need for the next season). This could be expected to progressively reduce heterozygosity to low levels. Thus, high heterozygosity and negative F index could result from positive selection for heterozygous loci, or from very low levels of self-pollination of individual plants. The latter could be the likely result of the monoecious sex habit of Faqqous (separate male and female flowers on same plant), and the very small number of female flowers found at a given time point on a single plant, favoring cross pollination between plants by honey bees and other pollinators. These pollinators may transfer pollen from adjacent fields, that will increase the diversity within individuals and decrease the diversity between populations, as was observed in the AMOVA. A Neighbor-Joining tree based on the same data also reflected the high within-population variation leading to less demarcated separation between populations (Fig. 10b). Still, the best tree obtained faithfully separated the four horticultural varieties, even better than the morphological tree (Fig. 4), that was based on few traits, compared to the extensive genomic sampling of the molecular tree. This confirms that the four main horticultural varieties have been selected over a rather long period and reflect true genetic divergence; AWG, a commercial Green Baladi Faqous recently bred in Natufa valley, clusters with White Baladi from the same area, from which it could have been derived. Also in the Sahouri group, the green accessions seem to stem out of the larger White Sahouri cluster. In the morphological tree, fruit color greatly influences tree structure, being a clear-cut trait, and the two green varieties cluster together against the two white ones (with few exceptions).

In a study of *Coffea* genetic diversity using DArT seq, Observed Heterozygosity values were much lower (0.124; Garavito et al. [40] compared to our average of 0.364, and F values were positive. In *Cicer*, He and uHe values (based on DArT markers) were low in the cultivated accessions (~ 0.15), and reached ~ 0.3 in wild accessions [35].

Populations are seldom reproductively isolated, and gene flow either by pollen or seed exchange can provide mixing. On the other hand, the positive correlation between geographic distance and genetic variation (Fig. 11) supports local evolution of landraces, and their adaptation over a long period of local selection, to the diverse environments found in Israel over short distances, e.g., 170–600 mm annual precipitation. PCoA analysis divided the samples in a way that largely reflects the geographic map, including the separation between Israel and the West Bank territories that have been administratively separated for the last decades. Moreover, the fact that a few climatic parameters were correlated with genetic variation, with specific quantitative traits, or with their coefficients of variation (Table 4), also supports a long-term local evolution of landraces in response to climatic factors. The high correlation of genetic distance with geographic distance suggests that gene flow is mediated by local agents such as pollinators and not by long distance agents such as human trade.

## Conclusions

Landraces in the surveyed area fall into four morphologically distinct horticultural varieties that are also genetically distinct. The extensive phenological-horticultural variation found in this collection, both within and between populations, suggests that selection for yield and related traits, such as femaleness and vigor, could result in yield improvement. Populations taken from individual grower field are highly heterozygous and polymorphic, in agreement with high rates of out-crossing between monoecious plants and significant gene flow between sites. Observed correlations between quantitative traits and climatic variables could suggest local adaptation of landraces to the diverse environment in which they evolved, and support our view of snake melon as an ancient link in melon domestication in the Near East. Our data stress the importance of preserving such germplasm, and demonstrate its potential for breeding better snake melons as an heirloom crop in our region.

## Additional file

Additional file 1: Omari et al. **Table S1.** Data on landraces used in this study and their respective collection sites. Table provides, for each population (collection site), the full set of geographic and climatic variables, morphological scores, and genetic diversity parameters that were calculated from the molecular data. (XLS 103 kb)
