# Correction to: Landraces of snake melon, an ancient Middle Eastern crop, reveal extensive morphological and DNA diversity for potential genetic improvement

**DOI:** 10.1186/s12863-018-0632-9

**Published:** 2018-07-12

**Authors:** Samer Omari, Yuri Kamenir, Jennifer I. C. Benichou, Sarah Pariente, Hanan Sela, Rafael Perl-Treves

**Affiliations:** 1 Mina and Everard Goodman Faculty of Life Sciences, Bar Ilan University, 5290002 Ramat Gan, Israel; 2 Department of Geography and Environment, Bar Ilan University, 5290002 Ramat Gan, Israel; 3 Cereal Crop Improvement Institute, Faculty of Life Sciences, Tel-Aviv University, 6997801 Tel Aviv, Israel

## Correction

Following publication of the original article [1], the authors reported the need for a more detailed acknowledgement of the source of the samples that were analyzed and their coordinates, which are discussed in the ‘Methods’ section of the article. This Correction provides an addition to the ‘Methods’ section, and a subsequently revised ‘Acknowledgements’ and ‘Availability of data and materials’ section.

**Addition to the ‘Methods’ section:** The seeds for 41 of the populations grown in the common garden experiment (composed of 434 individual accessions from 48 fields from 27 Palestinian villages) were provided by the research group of Prof. Ali-Shtayeh, Biodiversity and Environmental Research Center, Til, Nablus, Palestine, as part of MERC-USAID collaborative project TA-MOU-12- M32–016. The above 41 populations were described by Ali-Shtayeh et al. [2], and they cover all the populations in Table 1 except for populations MNZ, MZ2, AWG, AWW and samples GRG, GRW, MRA, FLT, FLI assembled by the present authors and not included in [2]. Data for each accession (village and district name, accession codes and common names, GPS coordinates) were provided to Perl-Treves, along with a soil sample of each field (below, “determination of soil type”).

In the original article [1], the research by Ali-Shtayeh et al. is cited as reference 16.

**Revised ‘Acknowledgements’ section:** The authors thank Prof. Ali-Shtayeh and his group at the Biodiversity and Environmental Research Center, Til, Nablus, Palestine, for providing, as part of MERC-USAID collaborative project TA-MOU-12- M32–016, the seeds of 41 populations for the present study. The provided samples represented 434 individual accessions from 48 fields from 27 Palestinian villages. This refers to all the populations listed in Table 1, except for populations MNZ, MZ2, AWG, AWW, and samples GRG, GRW, MRA, FLT, FLI, assembled by the present authors. The above 41 populations were described by Ali-Shtayeh et al. [16]. Data for each accession (village and district name, accession codes and common names, GPS coordinates) were also provided to Perl-Treves, along with a soil sample from each field. Part of the fruit pictures included in Fig. 1A was provided as well.

The authors thank Mr. Nabil Omari of the Extension services of the Israeli Ministry of agriculture for agronomic advice and Mr. David Levy for technical assistance.

**Revised ‘Availability of data and materials’ section:** Full phenotypic and genotypic data files that served to generate this study are deposited in the Israeli Genbank database https://igb.agri.gov.il/ and linked to the snake melon collection described in this study (samples 300,031–300,089). Seed samples for research purpose will be available from the Israeli Gene Bank.
